# Supplementary material for: Soluble Spike DNA Vaccine Provides Long-Term Protective Immunity against SARS-CoV-2 in Mice and Nonhuman Primates
Source: Vaccines (Basel). 2021 Mar 24;9(4):307. doi: 10.3390/vaccines9040307 (PMC8063950; doi:10.3390/vaccines9040307)
Supplement: Supplementary file 1 [file vaccines-09-00307-s001.pdf]

Supplymentary:

(a)

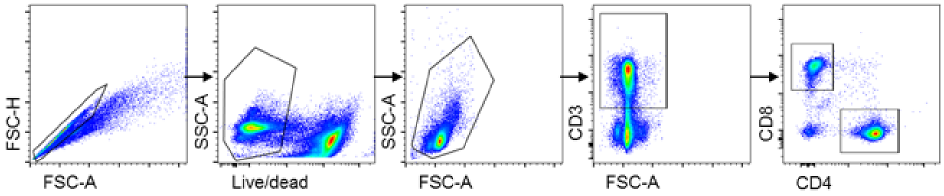

(b)

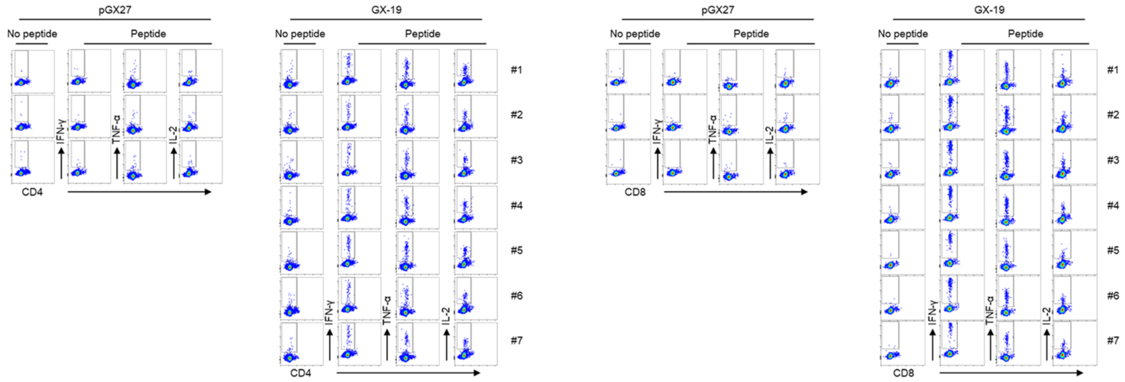

Figure 1. Flow cytometry panel to quantify SARS-CoV-2 S-specific mouse CD4⁺ or CD8⁺ T cells.

Mouse splenocytes were stimulated with specific peptide pools and then analyzed with multicolor flow cytometry to simultaneously detect SARS-CoV-2 S-specific expression of IFN-γ, TNF-α, and IL-2. Gating strategy to identify $CD4^+$ or $CD8^+$ T cells **(a)**. The representative plots show the frequencies of IFN-γ, TNF-α, IL-2 producing $CD4^+$ or $CD8^+$ T cells, respectively **(b)**.

(a)

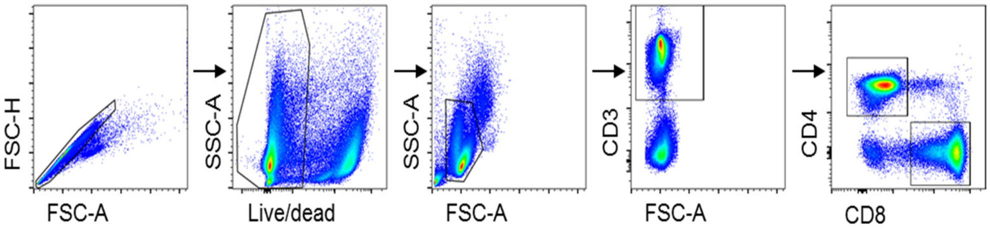

(**b**)

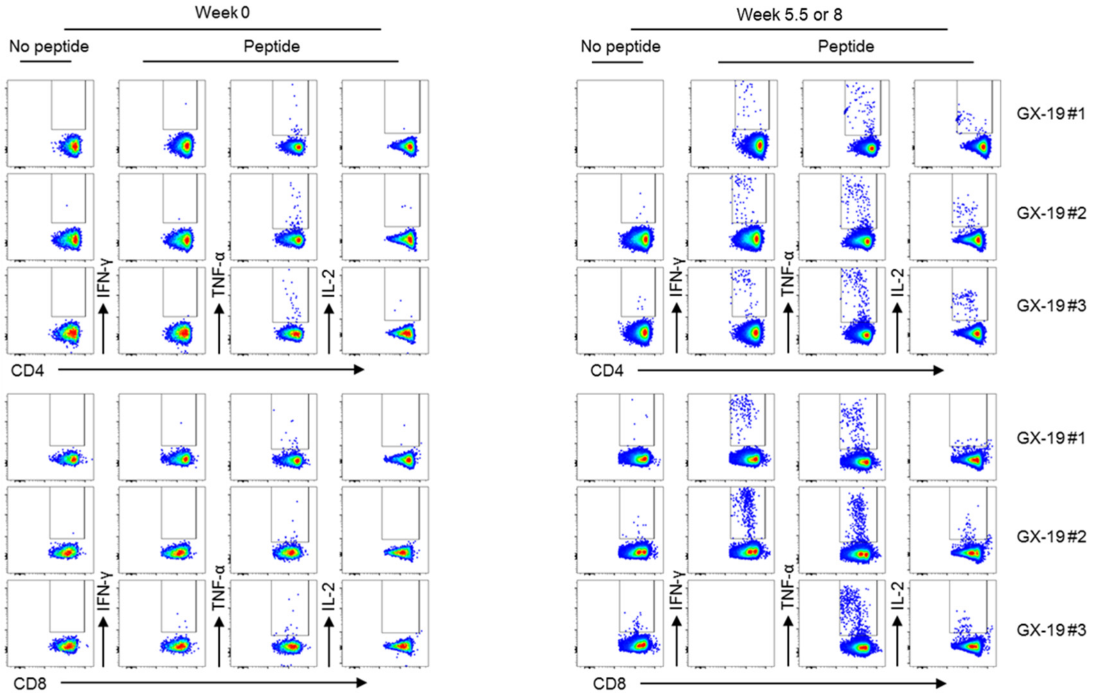

Figure 2. Flow cytometry panel to quantify SARS-CoV-2 S-specific NHP CD4+ or CD8+ T cells.

Cryopreserved PBMCs of GX-19 vaccinated macaques were stimulated with specific peptide pools and then analyzed with multicolor flow cytometry to simultaneously detect SARS-CoV-2 S-specific expression of IFN-γ, TNF-α, and IL-2. Gating strategy to identify CD4+ or CD8+ T cells (**a**). The representative plots show the frequencies of IFN-γ, TNF-α, IL-2 producing CD4+ or CD8+ T cells, respectively (**b**).

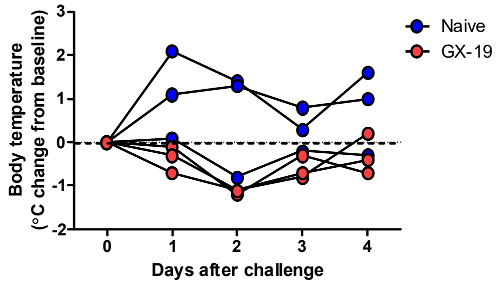

(**a**)

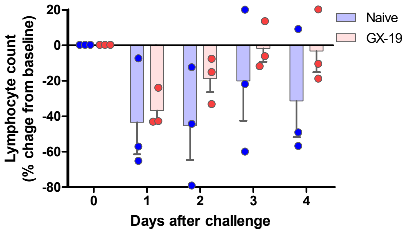

(**b**)

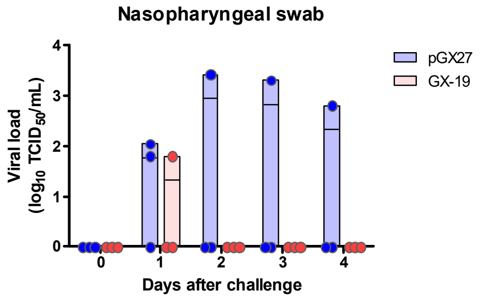

(**c**)

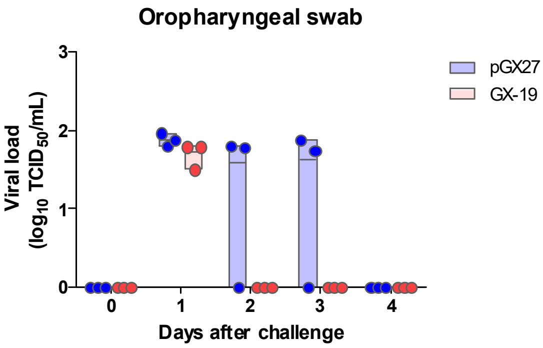

(**d**)

Figure 3. Clinical signs and $TCID_{50}$/mL in macaques challenged with SARS-CoV-2 after vaccination with GX-19.

Non-vaccinated and vaccinated macaques (*n*=3/group) were challenged with 2.7 x 10^7 $TCID_{50}$ SARS-CoV-2. After viral challenge, macaques were anesthetized for checking body temperature **(a)**, and blood lymphocyte count **(b)** at 0, 1, 2, 3, and 4 days post-infection (dpi). $TCID_{50}$/mL was measured in nasopharyngeal **(c)**, and oropharyngeal **(d)** at multiple time-points following challenge. *P* values determined by Mann-Whitney test;
